# Impact of thermal radiation and non-uniform heat flux on MHD hybrid nanofluid along a stretching cylinder

**DOI:** 10.1038/s41598-021-99800-0

**Published:** 2021-10-12

**Authors:** Aamir Ali, Tasmia Kanwal, Muhammad Awais, Zahir Shah, Poom Kumam, Phatiphat Thounthong

**Affiliations:** 1 Department of Mathematics, COMSATS University Islamabad, Attock Campus, Kamra Road, Attock, 43600 Pakistan; 2 Department of Mathematical Sciences, University of Lakki Marwat, Khyber Pakhtunkhwa, Lakki Marwat, 28420 Pakistan; 3 Fixed Point Research Laboratory, Fixed Point Theory and Applications Research Group, Center of Excellence in Theoretical and Computational Science (TaCS-CoE), Faculty of Science, King Mongkut’s University of Technology Thonburi (KMUTT), 126 Pracha Uthit Rd., Bang Mod, Thung Khru, Bangkok, 10140 Thailand; 4 Department of Medical Research, China Medical University Hospital, China Medical University, Taichung, 40402 Taiwan; 5 Renewable Energy Research Centre, Department of Teacher Training in Electrical Engineering, Faculty of Technical Education, King Mongkut’s University of Technology North Bangkok, 1518 Pracharat 1 Road, Bangsue, Bangkok, 10800 Thailand

**Keywords:** Engineering, Mathematics and computing, Nanoscience and technology

## Abstract

The current research investigates the thermal radiations and non-uniform heat flux impacts on magnetohydrodynamic hybrid nanofluid (CuO-Fe_2_O_3_/H_2_O) flow along a stretching cylinder, which is the main aim of this study. The velocity slip conditions have been invoked to investigate the slippage phenomenon on the flow. The impact of induced magnetic field with the assumption of low Reynolds number is imperceptible. Through the use of appropriate non-dimensional parameters and similarity transformations, the ruling PDE’s (partial differential equations) are reduced to set of ODE’s (ordinary differential equations), which are then numerically solved using Adams–Bashforth Predictor–Corrector method. Velocity and temperature fields with distinct physical parameters are investigated and explored graphically. The main observations about the hybrid nanofluid and non-uniform heat flux are analyzed graphically. A decrease in the velocity of the fluid is noted with addition of Hybrid nanofluid particles while temperature of the fluid increases by adding the CuO-Fe_2_O_3_ particles to the base fluid. Also, velocity of the fluid decreases when we incorporate the effects of magnetic field and slip. Raise in curvature parameter γ caused enhancement of velocity and temperature fields at a distance from the cylinder but displays opposite behavior nearby the surface of cylinder. The existence of heat generation and absorption for both mass dependent and time dependent parameters increases the temperature of the fluid.

## Introduction

Recently, many researchers attracted towards nano technology because of its vast applications in different fields. Nano fluids have different thermo-physical properties than their respective base fluids, which have poor ability to conduct heat. Nano fluid was firstly introduced by Choi^1^ in 1995 as a remedy to heat transfer enhancement. He found that nanofluid has greater thermal conductivity as compared to base fluid. Nanofluids are widely used in cancer therapeutics, nuclear reactor, refrigerators and also have many automobile and electronic applications. Buongiorno’s^2^ conducted a comprehensive study of convective transport in nanofluids. Khan and Pop^3^ investigated boundary layer flow of nanofluid across a stretched surface. They consider the thermophoresis effects and Brownian motion and solve the problem numerically. Nadeem and Lee^4^ analyze nanofluid flow via an exponentially stretched surface and use HAM to solve the problem analytically. Das et al.^5^ investigated the unsteady problem of nanofluid along stretching surface and used the shooting method in conjunction with the Runge–Kutta Fehlberg methodology to solve the problem numerically. The effects of radiation and varying fluid characteristics on unsteady boundary layer flow of a nanofluid are numerically discussed by Daba and Devaraj^6^. Awais et al.^7^ studied the polymeric material’s properties like nonlinear thermal radiation and solve the problem analytically by using HAM. Ali et al.^8^ used OHAM to solve the problem of three-dimensional Maxwell nanofluid across an exponentially extending surface. Later on, Ali et al.^9^ studied the nanofluid flow phenomenon for peristaltic flow with double diffusion. Poply and Vinita^10^ considered radiation and heat generation effects in their heat transfer analysis of nanofluid over stretching cylinder. Vinita et al.^11^ presented the two-components modeling of free stream velocity for MHD nanofluids over stretching cylinder. Jamshed et al.^12–14^ presented an optimal case study for evaluating unsteady nanofluid along a stretching surface, a mathematical model for heat transfer analysis of second grade nanofluid over a permeable flat surface, also done a comparative study of Williamson nanofluid by using Keller box method. Later on, Jamshed^15^ discussed the numerical investigations of impact of MHD on Maxwell nanofluid.

Now a day, a new category of nanofluids named hybrid nanofluids are emerging. Hybrid nanofluids are obtained by distributing two altered nano particles into base fluid. Hybrid nanofluids have considerable employment in different areas of heat transfer for example manufacturing process, medical, transport and defense. Hybrid nanofluid being advance nanofluid is utilized to further enhance the heat transfer rate. Sureh et al.^16^ inspected hybrid nanofluid for the first time practically and conclude that both thermal conductivity and viscosity of hybrid nanofluid can be increased with nanoparticles volume concentration. The experimental analysis of hybrid nanofluid is presented by Madhesh and Kalaiselvam^17^. By using and Ag-Mgo/Water hybrid nanofluid, Esfe et al.^18^ describe the experimental results on thermal conductivity and dynamics viscosity. Devi and Devi^19^ numerically discuss the effects of Cu- Al_2_O_3_/water hybrid nanofluid flow along a porous stretching surface. Hayat and Nadeem^20^ present the three-dimensional rotating flow of Ag-CuO/water hybrid nanofluid above a stretching surface. Sajid and Ali^21^ present critical review of the thermal conductivity of hybrid nanofluids. Jamshed and Aziz^22^ present the entropy analysis for TiO_2_-CuO/EG Casson hybrid nanofluid flow along a stretching surface and consider the Cattaneo-Christove heat flux model. Chamkha et al.^23^ examined the heat transfer analysis of MHD flow of hybrid nanofluid in a rotating system. Ellahi et al.^24^ investigated the slip effects on two-phase flow of hybrid nanofluid with Hafnium particles. Nawaz et al.^25^ considered hybrid nanofluid for improvement of thermal performance of ethylene glycol. Ali et al.^26^ performed a numerical analysis to present the effects of hybrid nanofluid on peristaltic flow by considering TiO_2_–Cu/H_2_O hybrid nanoparticles along with slip conditions. Mumraiz et al.^27^ presented the entropy generation analysis in MHD flow for Al_2_O_3_–Cu/H_2_O hybrid nanoparticles. Khan et al.^28^ discuss the second law analysis and present the analytical results for Al_2_O_3_-Ag/H_2_O hybrid nanofluid that is influenced by induced magnetic fields. Mourad et al.^29^ quantitatively represent the thermal aspects of Fe_3_O_4_-MWCNT/water hybrid nanofluid in a wavy channel numerically by using Galerkin finite element method. Jamshed et al.^30–32^ also presented a thermal case study of Cattaneo-Christov heat flux model on Williamson hybrid nanofluid by considering engine oil as base fluid, thermal expansion optimization of tangent hyperbolic hybrid nanofluid in solar aircraft, and shape effects of single-phase Williamson hybrid nanofluid Ag–Cu/EO flow over a stretching surface.

For the past years, many scholars examined heat transfer rate for boundary layer fluid flow owing to stretchable cylinder due to its applications in manufacturing and engineering procedures. Accordingly for the first time Wang^33^ presented the ambient fluid flow at rest on account of stretchable hollow cylinder. Cooling towers, crystal growing, wire drawing, cooling of electronic ships, paper and glass fiber production are some application fields of stretching cylinder^34^. The MHD boundary layer flow across a stretching cylinder was presented by Mukhopadhyay^35^. Poply et al.^36^ investigated laminar flow across a stretching cylinder. The heat transfer analysis of MHD boundary layer flow of ferro fluid through a stretching cylinder was studied by Qasim et al.^37^. Bilal et al.^38^ presented the effect of nanofluid along stretching cylinder. They deduced that for stretchable cylinder enhancement of temperature and velocity profiles are much greater as compared to stretching sheet. The stability analysis of MHD flow on a stretching cylinder was discussed by Poply et al.^39^. Ali et al.^40^ discuss the stretching cylinder analysis for third grade fluid with heat source/sink effects.

Effects of velocity slip have been considered by many researchers under various circumstances due to its broad practical interest. Slip flow takes place where the flow pressure is very weak. Slip boundary condition was first extracted by Maxwell^41^ and is widely implemented in current flow investigator. Slip flows have various implementations in medical fields for example in the polishing of simulated heart valves and polymeric technology. Ibrahim and Shankar^42^ investigated MHD boundary layer flow and discussed heat transfer analysis while taking slip conditions into account. By addressing non-uniform heat flux effects, Das et al.^43^ investigated slip phenomenon on MHD boundary layer flow of nanofluid over a vertical stretched sheet. In the presence of thermal radiation, Haq et al.^44^ examined the slip effects over stagnation point flow. The effects of velocity, temperature, and concentration slip on MHD nanofluid were discussed by Awais et al.^45^. Raza^46^ discussed the effects of thermal radiation on Casson fluid stagnation point flow under slip conditions. Ali et al.^47^ studied entropy generation on MHD peristaltic flow for two-phase nanofluid in the presence of slip effects. The slip effects on heat transfer study of nanofluid over a stretching cylinder are presented by Vinita and Poply^48^. Slip and hall effects on peristaltic flow of nanofluid with generalized complaint walls are shown by Awais et al.^49^. On a non-linear stretching cylinder, Vinita et al.^50^ investigate the velocity, temperature and concentration slip effects. Ali et al.^51^ discuss the impact of hall current and viscous dissipation on the slippage phenomenon in MHD peristaltic flow.

This work aims to determine the problem of hybrid nanofluid flow along a stretching cylinder. Hybrid nanofluids have been identified as prospective fluids and have gained considerable attraction from researchers, due to their wide range of applications in the medical and engineering sector. We have considered Copper Oxide (CuO) and Ferrous Oxide (Fe_2_O_3_) as nanometer size particles with water as a base fluid. Non-uniform heat flux and thermal radiation effects have been taken into account. The velocity slip condition will be invoked to study the slip effects on the flow. The mathematical modeling is carried out by using the continuity, momentum and energy equation. These governing equations are PDE’s (partial differential equations), thus they are transformed into set of ODE’s (ordinary differential equations) that may be solved numerically. Plots of different physical quantities were created to gain a better understanding of the subject under consideration.

## Mathematical formulation

**Figure 1:**
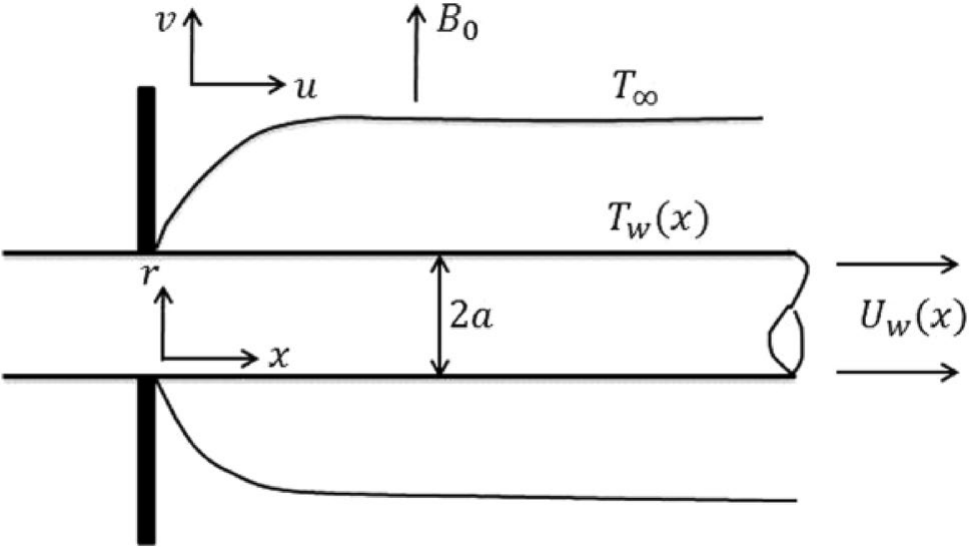
Geometry of the problem.

We have considered a two-dimensional, axisymmetric, unsteady, incompressible, boundary layer hybrid nanofluid flow past a stretchable cylinder having radius *b* in existence of uniform magnetic field. For this study, we have considered Copper Oxide (CuO) and Ferrous Oxide (Fe_2_O_3_) nanometer size particles with water as a base fluid. Cylindrical coordinate system has been considered with $(x,r)$-axes are taken along the cylinder’s radial and axial direction respectively. A uniform magnetic field of strength $B_{0}$ is used in the radial direction. Magnetic field produced by induction is insignificant as compare to applied magnetic field with the supposition of small magnetic Reynolds number. The cylinder is being stretched in the axial direction, and the cylinder’s stretching velocity is $U_{w} = U_{0} \left( \frac{x}{l} \right)$, where $U_{0}$ is the associated velocity and *l* is the characteristic length. The surface temperature $T_{w} \left( x \right)$ is believed to be higher than the ambient temperature $T_{\infty } .$ The illustrative diagram is displayed in Fig. 1.

The vector form of the governing equations is:

1 $$\nabla \cdot {\mathbf{V}} = 0,$$

2 $$\rho_{hnf} \frac{{d{\mathbf{V}}}}{dt} = \mu_{hnf} \nabla \cdot {\mathbf{T}} + {\mathbf{J}} \times {\mathbf{B}},$$

3 $$\left( {\rho c_{p} } \right)_{hnf} \frac{dT}{{dt}} = \kappa_{hnf} \nabla^{2} T - \nabla q_{r} + q^{{{\prime \prime \prime }}} .$$

where ${\mathbf{V}} = \left[ {v\left( {r,x} \right),0,u\left( {r,x} \right)} \right]$ is the velocity field in cylindrical coordinate system, $\frac{d}{dt}$ is the material time derivative, ${\mathbf{J}} \times {\mathbf{B}}$ is the Lorentz force vector calculated from ohm’s law, $\rho_{hnf}$ is the hybrid nanofluid density, $\mu_{hnf}$ is the hybrid nanofluid viscosity, $\left( {\rho c_{p} } \right)_{hnf}$ is the hybrid nanofluid heat capacity, $T$ is the fluids temperature and $\kappa_{hnf}$ is the hybrid nanofluid thermal conductivity. $q_{r}$ is the nonlinear radiative heat flux^52^, which may be derived using Rossland’s approximation and is given by:

$$q_{r} = - \frac{{4\sigma^{*} }}{{3k^{*} }}\frac{{\partial T^{4} }}{\partial r},$$

where $\sigma^{*}$ is the Stefan Boltzmann coefficient and $k^{*}$ is the mean absorption coefficient. Utilizing Taylor’s expansion of $T^{4}$ around $T_{\infty }$ which is the ambient temperature and ignoring higher order terms, we get,

4 $$\begin{aligned} & T^{4} \cong 4T_{\infty }^{3} T - 3T_{\infty }^{4} \\ & q_{r} = - \frac{{16\sigma^{*} }}{{3k^{*} }}T_{\infty }^{3} \frac{\partial T}{{\partial r}}, \\ \end{aligned}$$

$q^{{{\prime \prime \prime }}}$ is the non-uniform heat flux and is defined as^27^:

5 $$q^{{{\prime \prime \prime }}} = \frac{{\kappa_{hnf} U_{w} }}{{x\nu_{hnf} }}\left[ {X^{*} \left( {T_{w} - T_{\infty } } \right)f^{{\prime }} + Y^{*} \left( {T - T_{\infty } } \right)} \right]$$

Here, $X^{*}$ and $Y^{*}$ are space dependent and time dependent heat source and heat sink parameters. $X^{*} > 0$, $Y^{*} > 0$ symbolizes heat source and $X^{*} < 0, \, Y^{*} < 0$ symbolizes heat sink. Under the given assumptions, conservations laws of mass, momentum and energy Eqs. (1)–(3), in the presence of thermal radiation (4) and non-uniform heat generation/absorption (5) and using boundary layer approximations takes the following form^36,37^:

6 $$\frac{\partial }{\partial x}\left( {ru} \right) + \frac{\partial }{\partial r}\left( {rv} \right) = 0,$$

7 $$\rho_{hnf} \left( {u\frac{\partial u}{{\partial x}} + v\frac{\partial u}{{\partial r}}} \right) = \mu_{hnf} \left( {\frac{{\partial^{2} u}}{{\partial r^{2} }} + \frac{1}{r}\frac{\partial u}{{\partial r}}} \right) - \sigma_{hnf} {\text{B}}_{{0}}^{{2}} u,$$

8 $$\begin{aligned} \left( {\rho c_{p} } \right)_{hnf} \left( {u\frac{\partial T}{{\partial x}} + v\frac{\partial T}{{\partial r}}} \right) = & \kappa_{hnf} \left( {\frac{{\partial^{2} T}}{{\partial r^{2} }} + \frac{1}{r}\frac{\partial T}{{\partial r}}} \right) + \frac{{16\sigma^{*} T_{\infty }^{3} }}{{3k^{*} }}\frac{{\partial^{2} T}}{{\partial r^{2} }} \\ & \quad + \frac{{\kappa_{hnf} U_{w} }}{{x\nu_{hnf} }}\left[ {X^{*} \left( {T_{w} - T_{\infty } } \right)f^{\prime} + Y^{*} \left( {T - T_{\infty } } \right)} \right]. \\ \end{aligned}$$

The suitable boundary conditions affiliated with considered flow are:

9 $$\begin{array}{*{20}l} {u = \, U_{w} { + }\mu_{hnf} \delta \frac{\partial u}{{\partial r}}{,}v = \, 0{,}T = T_{w} } \hfill & {{\text{at}}\;r = b,} \hfill \\ {u \to 0{,}T \to T_{\infty } } \hfill & {{\text{as}}\;r \to \infty .} \hfill \\ \end{array}$$

Let us specify dimensionless parameters as^36,37^:

10 $$\eta = \frac{{r^{2} - b^{2} }}{2b}\left( {\frac{{U_{w} }}{{\nu_{f} x}}} \right)^{1/2} ,\,\psi = b\left( {\nu_{f} U_{w} x} \right)^{1/2} f\left( \eta \right),\,\theta \left( \eta \right) = \frac{{T - T_{\infty } }}{{T_{w} - T_{\infty } }}.$$

Equation (6) is instinctively fulfilled with stream function $\psi$ as

$$u = \frac{1}{r}\frac{\partial \psi }{{\partial r}}\;{\text{and}}\;v = - \frac{1}{r}\frac{\partial \psi }{{\partial x}}.$$

Substituting Eq. (10) in Eqs. (7)–(9) we gain the resulting dimensionless form of equations:

11 $$\begin{aligned} & \left( {1 + 2\gamma \eta } \right)f^{{{\prime \prime \prime }}} + 2\gamma f^{{\prime \prime }} - \left( {1 - \phi_{1} } \right)^{2.5} \left( {1 - \phi_{2} } \right)^{2.5} \left[ {\left( {1 - \phi_{2} } \right)\left\{ {\phi_{1} \left( {\frac{{\rho_{{s_{1} }} }}{{\rho_{f} }}} \right) + \left( {1 - \phi_{1} } \right)} \right\} + \phi_{2} \left( {\frac{{\rho_{{s_{2} }} }}{{\rho_{f} }}} \right)} \right]\left( {f^{{{\prime }2}} - ff^{{\prime \prime }} } \right) \\ & \quad - \left( {1 - \phi_{1} } \right)^{2.5} \left( {1 - \phi_{2} } \right)^{2.5} \frac{{\sigma_{hnf} }}{{\sigma_{f} }}Mf^{{\prime }} = 0, \\ \end{aligned}$$

12 $$\begin{aligned} & \left( {\frac{{\kappa_{hnf} }}{{\kappa_{f} }} + Rd} \right)\left( {1 + 2\gamma \eta } \right)\theta^{{\prime \prime }} + \left( {2\frac{{\kappa_{hnf} }}{{\kappa_{f} }} + Rd} \right)\gamma \theta^{{\prime }} + \Pr \left[ {\left( {1 - \phi_{2} } \right)\left\{ {\phi_{1} \frac{{\left( {\rho c_{p} } \right)_{{s_{1} }} }}{{\left( {\rho c_{p} } \right)_{f} }} + \left( {1 - \phi_{1} } \right)} \right\} + \phi_{2} \frac{{\left( {\rho c_{p} } \right)_{{s_{2} }} }}{{\left( {\rho c_{p} } \right)_{f} }}} \right]f\theta^{{\prime }} \\ & \quad + \frac{{\kappa_{hnf} }}{{\kappa_{f} }}\left( {1 - \phi_{1} } \right)^{2.5} \left( {1 - \phi_{2} } \right)^{2.5} \left[ {\left( {1 - \phi_{2} } \right)\left\{ {\phi_{1} \left( {\frac{{\rho_{{s_{1} }} }}{{\rho_{f} }}} \right) + \left( {1 - \phi_{1} } \right)} \right\} + \phi_{2} \left( {\frac{{\rho_{{s_{2} }} }}{{\rho_{f} }}} \right)} \right]\left( {X^{*} f^{{\prime }} + Y^{*} \theta } \right) = 0. \\ \end{aligned}$$

13 $$\begin{array}{*{20}l} {f = 0,\;f^{{\prime }} = 1 + \frac{\beta }{{\left( {1 - \phi_{1} } \right)^{2.5} \left( {1 - \phi_{2} } \right)^{2.5} }}f^{{\prime \prime }} ,\theta = 1} \hfill & {{\text{at}}\;\eta = 0} \hfill \\ {f^{{\prime }} \to 0,\;\theta \to 0} \hfill & {{\text{as}}\;\eta \to \infty .} \hfill \\ \end{array}$$

The non-dimensional quantities existing in the Eqs. (11)–(13) are magnetic term *M*, Prandtl number *Pr*, curvature $\gamma$, radiation *Rd* and slip parameter $\beta$ defined as:

14 $$M = \frac{{\sigma_{hnf} B_{0}^{2} l}}{{\rho_{f} U_{0} }},\;\beta = \delta \mu_{f} \left( {\frac{{U_{0} }}{{l\nu_{f} }}} \right)^{1/2} ,\;Rd = \frac{{4\sigma^{*} T_{\infty }^{3} }}{{\kappa_{f} k^{*} }},\;\gamma = \left( {\frac{{l\nu_{f} }}{{U_{0} b^{2} }}} \right)^{\frac{1}{2}} {,}\;\Pr = \frac{{\mu_{f} \left( {\rho c_{p} } \right)_{f} }}{{\rho_{f} \kappa_{f} }}.$$

The skin friction coefficient and the Nusselt number are two important physical quantities, which are defined as follows:

15 $$C_{f} = \frac{{\mu_{hnf} }}{{\rho_{f} U_{w}^{2} }}\left( {\frac{\partial u}{{\partial r}}} \right)_{r = b} , \, Nu_{x} = - \frac{{x\kappa_{hnf} }}{{\kappa_{f} \left( {T_{w} - T_{\infty } } \right)}}\left( {\frac{\partial T}{{\partial r}}} \right)_{r = b} ,$$

Using the similarity variables Eq. (10) and stream function definitions into Eq. (15), we drive the following non-dimensional form of skin friction coefficient and Nusselt number:

16 $${\text{Re}}_{x}^{1/2} C_{f} = \frac{{\mu_{hnf} }}{{\mu_{f} }}f^{{\prime \prime }} \left( 0 \right),\;{\text{Re}}_{x}^{ - 1/2} Nu_{x} = - \frac{{\kappa_{hnf} }}{{\kappa_{f} }}\theta^{{\prime }} \left( 0 \right),$$

where ${\text{Re}}_{x} = U_{w} x/\nu_{f}$ is the local Reynolds number.

## Thermophysical properties

Table 1 displays the experimental relationships of hybrid nanofluid based on different thermal properties while Table 2 indicates the computational values for thermo-physical characteristics of nanoparticles and fluid yield to find the computational values of hybrid nanofluid properties.

**Table 1:** Empirical relations for thermophysical characteristics of hybrid nanofluid^26,27^.

Properties	Expressions for Hybrid nanofluid
Density	$\frac{{\rho_{hnf} }}{{\rho_{f} }} = \left( {1 - \phi_{2} } \right)\left[ {\phi_{1} \left( {\frac{{\rho_{{s_{1} }} }}{{\rho_{f} }}} \right) + \left( {1 - \phi_{1} } \right)} \right] + \phi_{2} \left( {\frac{{\rho_{{s_{2} }} }}{{\rho_{f} }}} \right)$
Viscosity	$\frac{{\mu_{hnf} }}{{\mu_{f} }} = \frac{1}{{\left( {1 - \phi_{1} } \right)^{2.5} \left( {1 - \phi_{2} } \right)^{2.5} }}$
Heat capacity	$\frac{{\left( {\rho c_{p} } \right)_{hnf} }}{{\left( {\rho c_{p} } \right)_{f} }} = \left( {1 - \phi_{2} } \right)\left[ {\phi_{1} \frac{{\left( {\rho c_{p} } \right)_{{s_{1} }} }}{{\left( {\rho c_{p} } \right)_{f} }} + \left( {1 - \phi_{1} } \right)} \right] + \phi_{2} \frac{{\left( {\rho c_{p} } \right)_{{s_{2} }} }}{{\left( {\rho c_{p} } \right)_{f} }},$
Thermal conductivity	$$\begin{gathered} \kappa_{hnf} = \frac{{\kappa_{{s_{2} }} + \left( {n - 1} \right)\kappa_{nf} - \left( {n - 1} \right)\phi_{2} \left( {\kappa_{nf} - \kappa_{{s_{2} }} } \right)}}{{\kappa_{{s_{2} }} + \left( {n - 1} \right)\kappa_{nf} + \phi_{2} \left( {\kappa_{nf} - \kappa_{{s_{2} }} } \right)}}\kappa_{nf} , \hfill \\ {\text{where}}\,\,\frac{{\kappa_{nf} }}{{\kappa_{f} }} = \frac{{\kappa_{{s_{1} }} + \left( {n - 1} \right)\kappa_{f} - \left( {n - 1} \right)\phi_{1} \left( {\kappa_{f} - \kappa_{{s_{1} }} } \right)}}{{\kappa_{{s_{1} }} + \left( {n - 1} \right)\kappa_{f} + \phi_{1} \left( {\kappa_{f} - \kappa_{{s_{1} }} } \right)}} \hfill \\ \end{gathered}$$
Electric conductivity	$$\begin{gathered} \sigma_{hnf} = \frac{{\sigma_{{s_{2} }} + 2\sigma_{nf} - 2\phi_{2} \left( {\sigma_{nf} - \sigma_{{s_{2} }} } \right)}}{{\sigma_{{s_{2} }} + 2\sigma_{nf} + \phi_{2} \left( {\sigma_{nf} - \sigma_{{s_{2} }} } \right)}}\sigma_{nf} , \hfill \\ {\text{where}}\,\,\frac{{\sigma_{nf} }}{{\sigma_{f} }} = \frac{{\sigma_{{s_{1} }} + 2\sigma_{f} - 2\phi_{1} \left( {\sigma_{f} - \sigma_{{s_{1} }} } \right)}}{{\sigma_{{s_{1} }} + 2\sigma_{f} + \phi_{1} \left( {\sigma_{f} - \sigma_{{s_{1} }} } \right)}}. \hfill \\ \end{gathered}$$

**Table 2:** Computational values for thermophysical characteristics of base fluid and nanoparticles^2,10^.

Physical properties	H_2_O	Fe_2_O_3_ (ϕ_1_)	CuO (ϕ_2_)
ρ (kgm^−3^)	997.1	3970	6500
*c*_*p*_ (J Kg^−1^ K^−1^)	4179	765	531.8
κ (Wm^−1^ K^−1^)	0.613	40	0.85
σ (Ω^−1^ m^−1^)	0.05	35 × 10^6^	59.6 × 10^6^

## Numerical procedure

In this portion we have presented the numerical strategy which is utilized for determining the solution of our system of non-dimensional equations subject to the given boundary conditions. For this purpose we have utilized the numerical approach Adams–Bashforth Predictor–Corrector method, which is a linear multistep method. Multistep methods try to enhance the efficiency preserving and utilizing the information from prior phases rather than discording it and thus refer to several previous points and derivative values. It works in two steps, first we use Adams–Bashforth method as prediction step which calculates a rough approximation of the desired quantity. Secondly, we use Adams–Moulton method as corrector step which refines the initial approximation. In Adams–Bashforth technique we established first order system of equations along with boundary conditions (13) in terms of *f*(*η*) and *θ*(*η*) from dimensionless Eqs. (11)–(12). First order system for *f*(*η*) is framed as:

$$\begin{aligned} & f_{1} = f^{{\prime }} ,f_{2} = f_{1}^{{\prime }} ,f_{3} = f_{2}^{{\prime }} \\ & f_{3} = f_{2}^{{\prime }} = - \frac{1}{{\left( {1 + 2\gamma \eta } \right)}}\left[ {A_{1} A_{2} \left( {f_{1}^{2} - ff_{2} } \right) + A_{1} A_{3} Mf_{1} + 2\gamma f_{2} } \right] \\ \end{aligned}$$

and temperature equation *θ*(*η*) as:

$$\begin{aligned} & \theta_{1} = \theta {\prime },\theta_{2} = \theta_{1}^{{\prime }} \\ & \theta_{2} = \theta_{1}^{{\prime }} = - \frac{1}{{\left( {A_{5} + Rd} \right)\left( {1 + 2\gamma \eta } \right)}}\left[ {\left\{ {\left( {2A_{5} + Rd} \right)\gamma + A_{4} \Pr f} \right\}\theta_{1} + A_{1} A_{2} A_{5} \left( {X^{*} f^{{\prime }} + Y^{*} \theta } \right)} \right]. \\ \end{aligned}$$

The suitable boundary conditions for *f*(*η*) and *θ*(*η*) are

$$\begin{gathered} f\left( 0 \right) = 0, \, f_{1} \left( 0 \right) = 1 + \frac{\beta }{{A_{1} }}f_{2} \left( 0 \right), \, \hfill \\ \, f_{1} \left( 1 \right) = 0,\,\,\,\,\theta \left( 0 \right) = 1, \, \theta \left( 1 \right) = 0. \hfill \\ \end{gathered}$$

where

$$\begin{aligned} & A_{1} = \left( {1 - \phi_{1} } \right)^{2.5} \left( {1 - \phi_{2} } \right)^{2.5} , \\ & A_{2} = \left[ {\left( {1 - \phi_{2} } \right)\left\{ {\phi_{1} \left( {\frac{{\rho_{{s_{1} }} }}{{\rho_{f} }}} \right) + \left( {1 - \phi_{1} } \right)} \right\} + \phi_{2} \left( {\frac{{\rho_{{s_{2} }} }}{{\rho_{f} }}} \right)} \right], \\ & A_{3} = \frac{{\sigma_{hnf} }}{{\sigma_{f} }}, \\ & A_{4} = \left[ {\left( {1 - \phi_{2} } \right)\left\{ {\phi_{1} \frac{{\left( {\rho c_{p} } \right)_{{s_{1} }} }}{{\left( {\rho c_{p} } \right)_{f} }} + \left( {1 - \phi_{1} } \right)} \right\} + \phi_{2} \frac{{\left( {\rho c_{p} } \right)_{{s_{2} }} }}{{\left( {\rho c_{p} } \right)_{f} }}} \right], \\ & A_{5} = \frac{{\kappa_{hnf} }}{{\kappa_{f} }}. \\ \end{aligned}$$

Acquired differential system for *f*(*η*) and *θ*(*η*) are generally represented as follows:

$$\begin{array}{*{20}l} {\frac{df}{{d\eta }} = q\left( {\eta ,f} \right),} \hfill & {f\left( {\eta_{0} } \right) = f_{0} } \hfill \\ {\frac{d\theta }{{d\eta }} = q\left( {\eta ,\theta } \right),} \hfill & {\theta \left( {\eta_{0} } \right) = \theta_{0} .} \hfill \\ \end{array}$$

**Figure 2:**
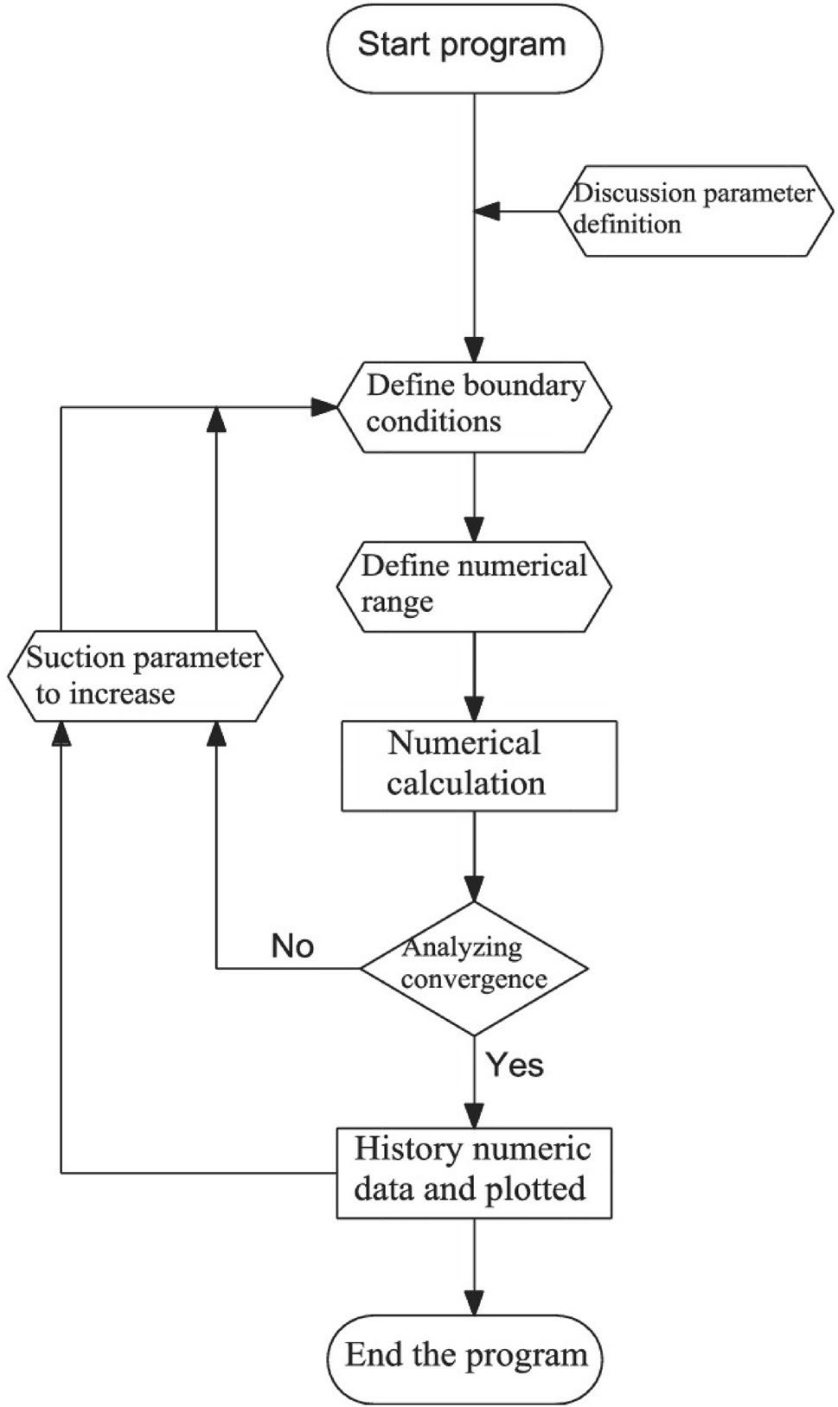
Flow chart of numerical scheme.

The general expression for two step Adams–Bashforth approach for *f*(*η*) and *θ*(*η*) are given respectively as follows

$$\begin{aligned} & f_{k + 1} = f_{k} + \frac{h}{2}\left[ {3q\left( {\eta_{k} ,f_{k} } \right) - q\left( {\eta_{k - 1} ,f_{k - 1} } \right)} \right] \\ & \theta_{k + 1} = \theta_{k} + \frac{h}{2}\left[ {3q\left( {\eta_{k} ,\theta_{k} } \right) - q\left( {\eta_{k - 1} ,\theta_{k - 1} } \right)} \right], \\ \end{aligned}$$

where *h* is a step size parameter. The flow chart of the scheme is given below in Fig. 2:

## Graphical analysis

**Figure 3:**
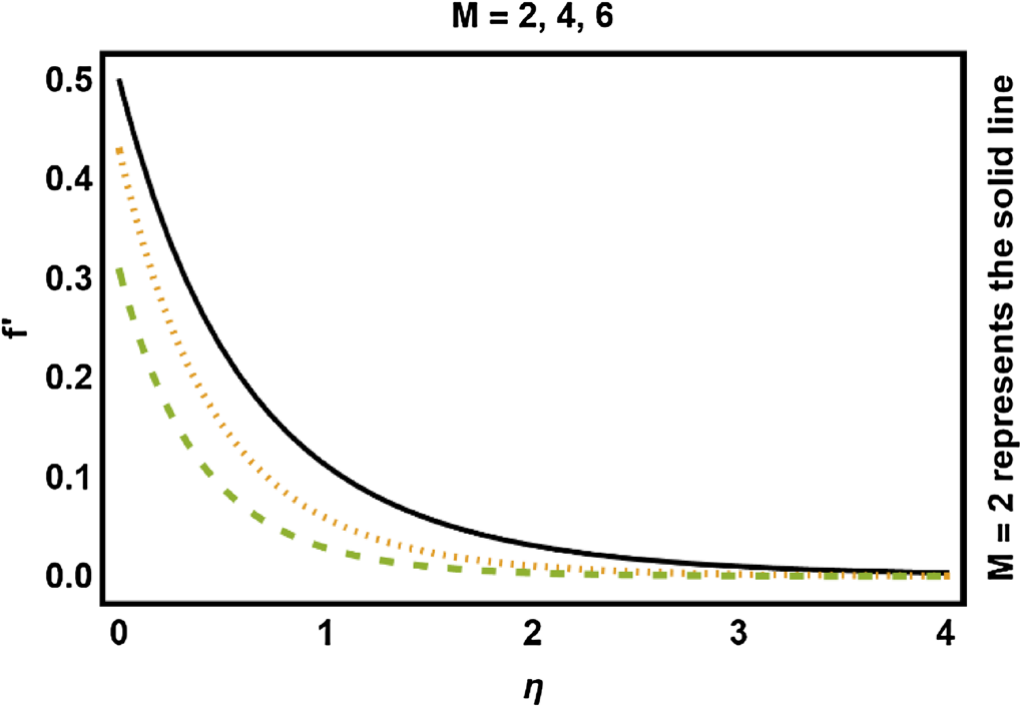
Magnetic effects on velocity field.

**Figure 4:**
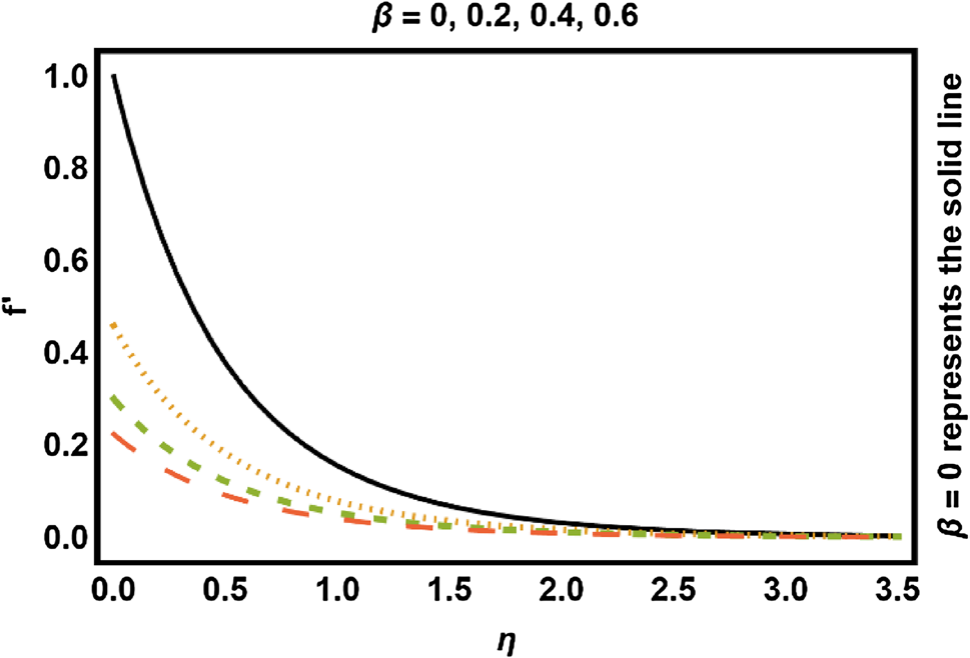
Slip effects on velocity field.

**Figure 5:**
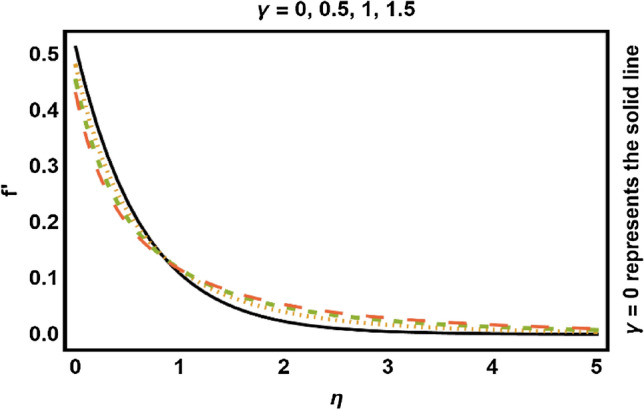
$\gamma$ on velocity field.

**Figure 6:**
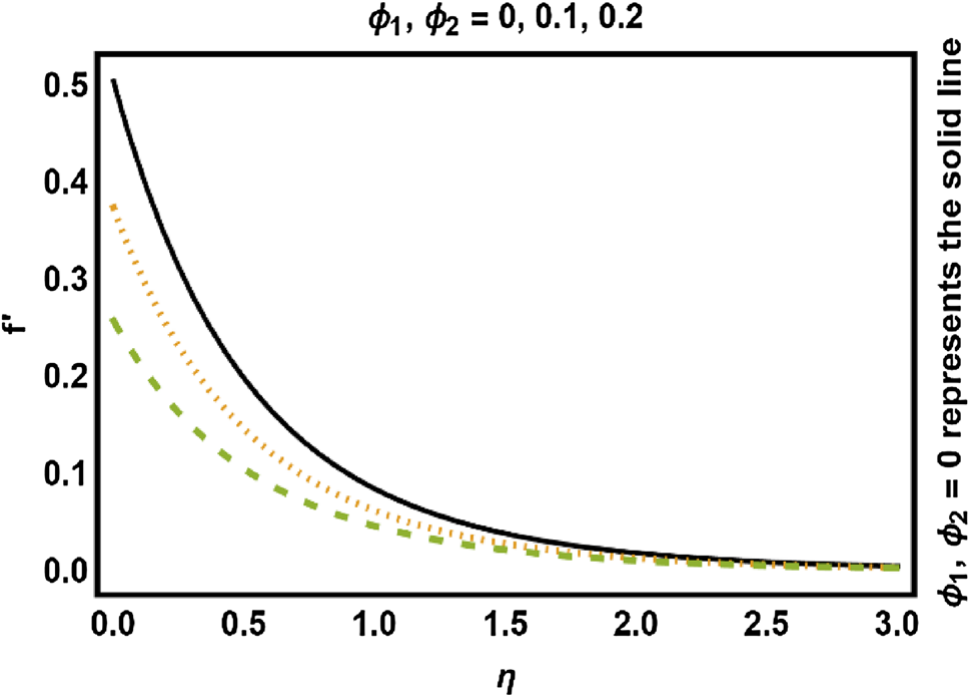
*ϕ*_1_, *ϕ*_2_ on velocity field.

**Figure 7:**
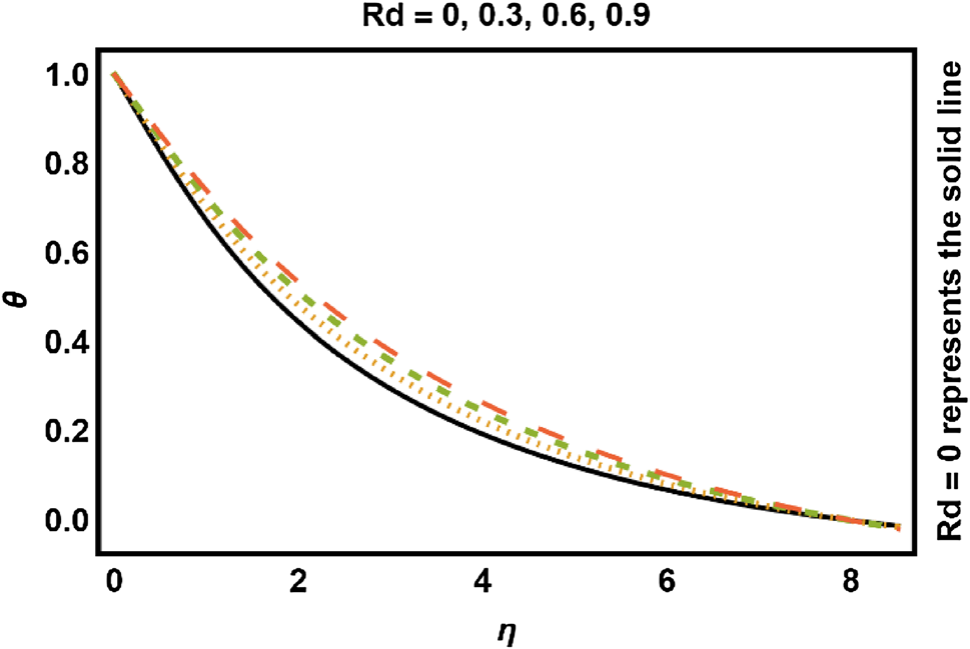
*Rd* on temperature field.

**Figure 8:**
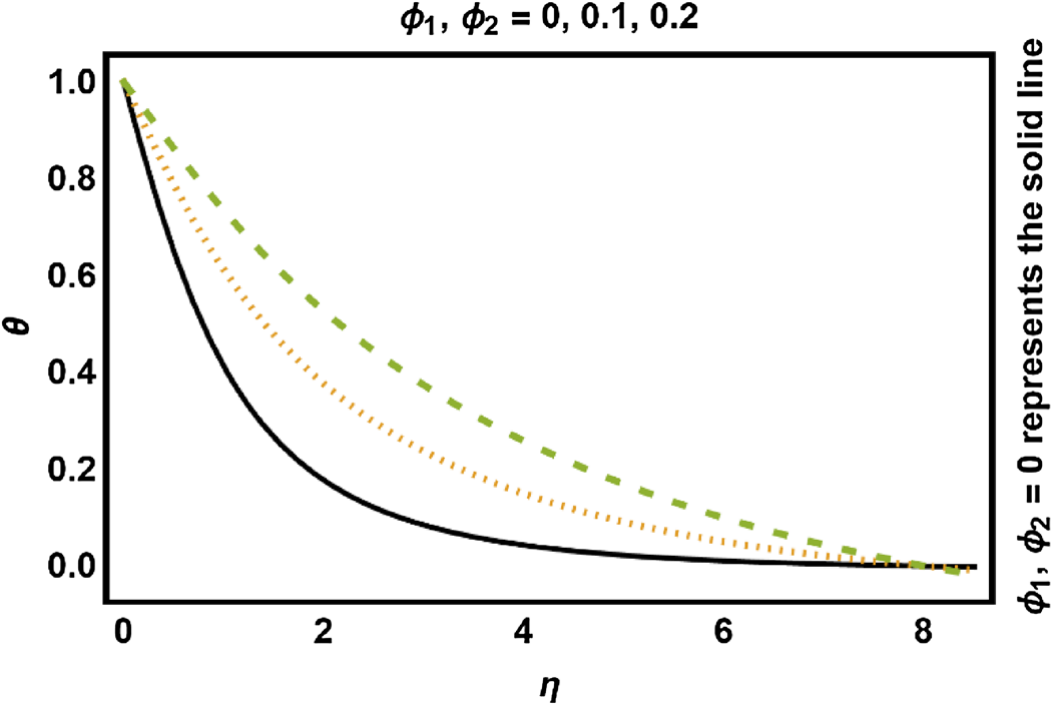
*ϕ*_1_, *ϕ*_2_ on temperature field.

**Figure 9:**
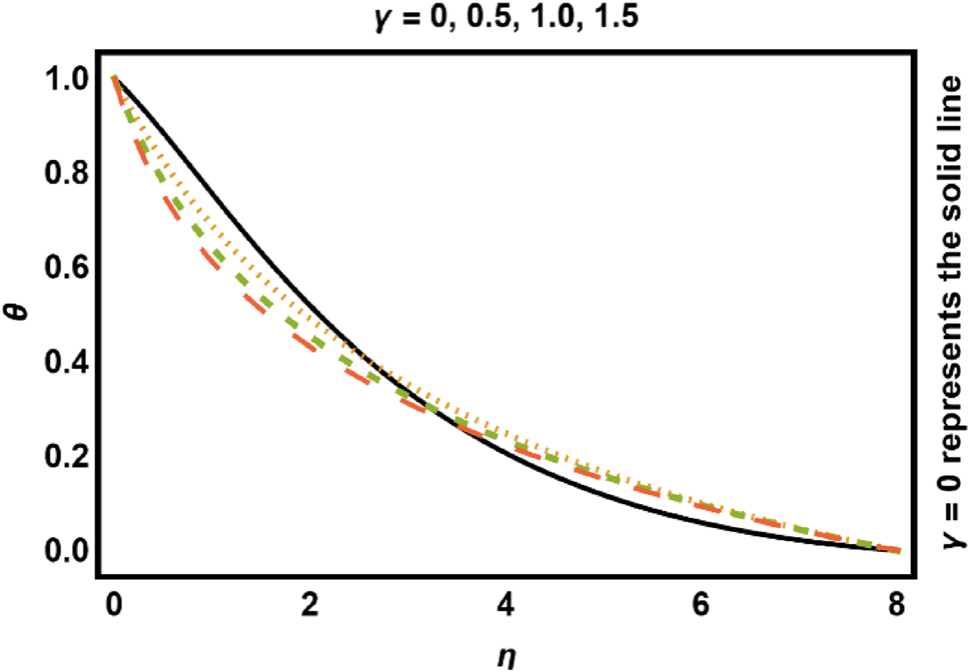
$\gamma$ on fluids temperature.

**Figure 10:**
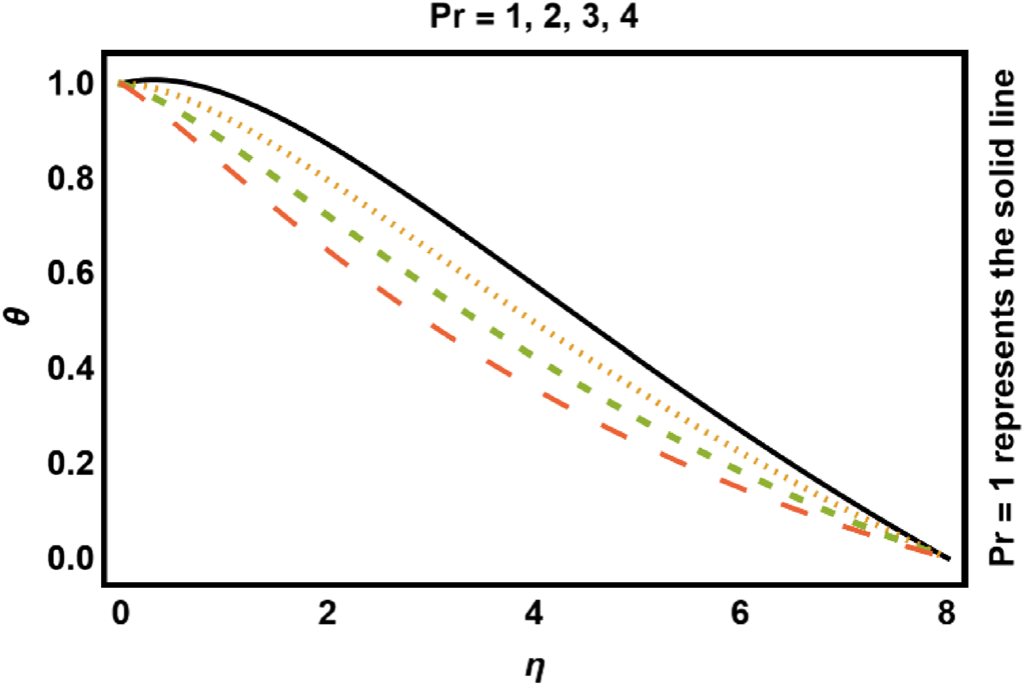
*Pr* on temperature profile.

**Figure 11:**
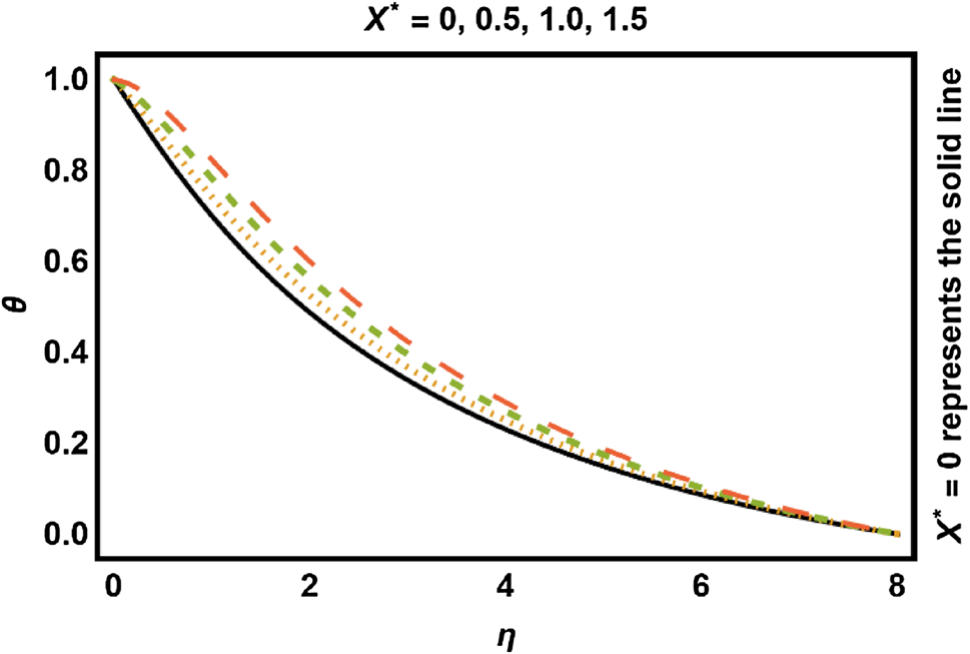
$X^{*} > 0$ on temperature field.

**Figure 12:**
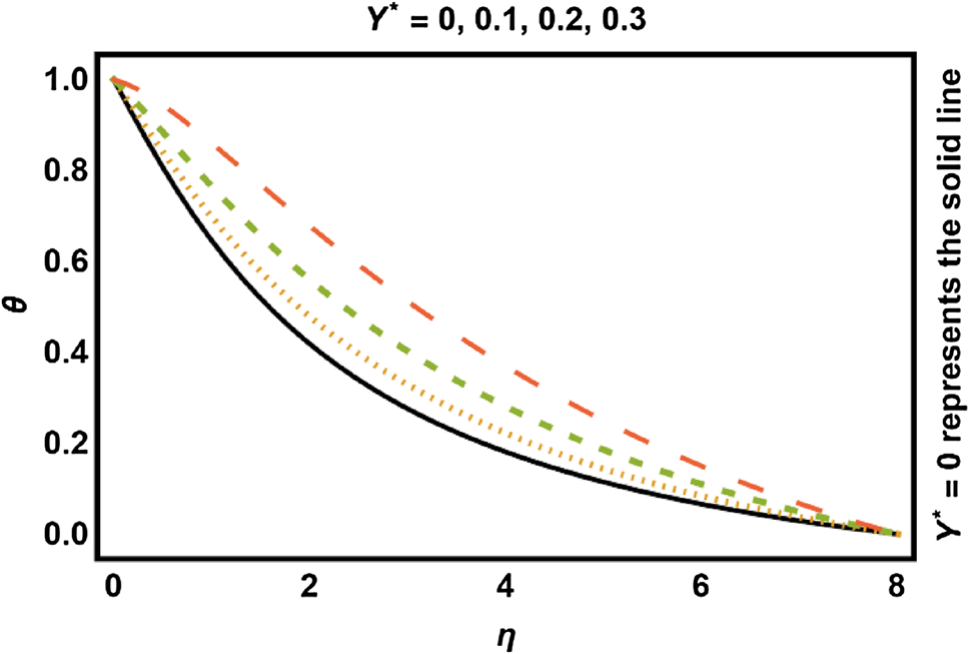
$Y^{*} > 0$ on temperature field.

**Figure 13:**
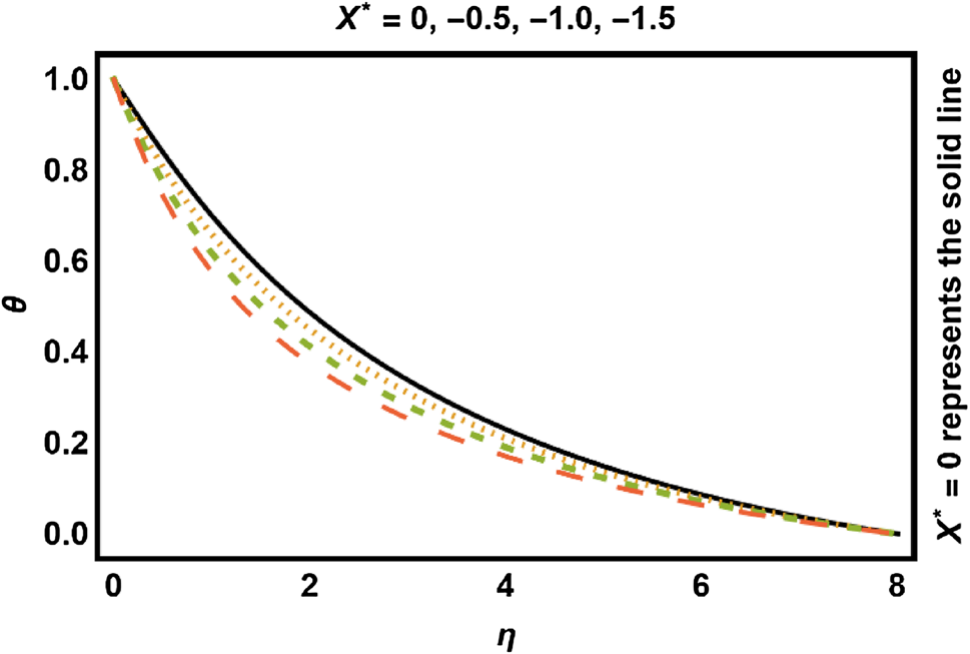
$X^{*} < 0$ on temperature field.

**Figure 14:**
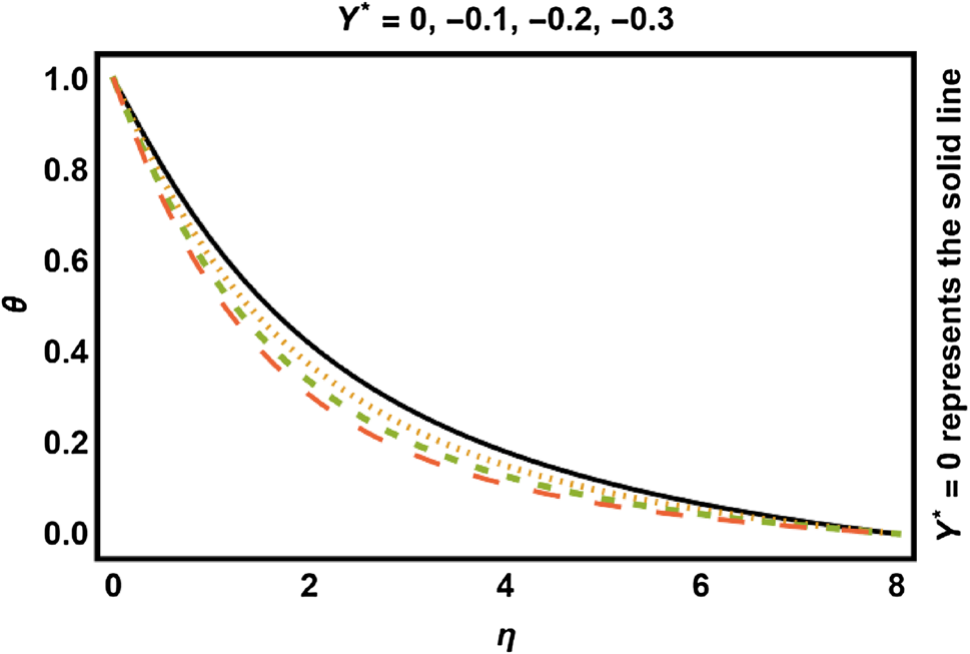
$Y^{*} < 0$ on temperature field.

In this portion, we have presented the impact of distinct quantities on the flow profiles. Equations (11)–(13) have been determined by utilizing numerical technique. Physical changes in velocity and temperature fields against distinct parameters are drafted in Figs. 3, 4, 5, 6, 7, 8, 9, 10, 11, 12, 13, 14. These distinct parameters are radiation, magnetic, slip and heat generation/absorption parameters along with Prandtl number *Pr* etc. The consequences of magnetic effects on velocity field have been expressed in Fig. 3. It is shown that velocity field decays with increment in *M*. Physically the Lorentz force act as a decelerating agent which reduces the fluid speed and momentum boundary layer thickness. Consequently higher values of *M*, strengthens the resistive force and that opposing the magnetic forces with dominant retarding effects and for that reason, *M* has shown a decreasing behavior on the velocity of the fluid. Increment in *β* deescalates the velocity field along with momentum boundary layer thickness as shown in Fig. 4. Due to influence of slip, fluid velocity nearby the stretchable surface is no more equivalent to the velocity of stretching cylinder. Increase in *β* causes slip velocity to increase hence speed of the fluid reduces. The reason behind this is that pulling of stretchable surface may only be slightly transformed to the fluid.

Figure 5 illustrated the influence of curvature parameter $\gamma$ on velocity field. It is depicted that velocity field reduces close to the surface of cylinder and elevates distance from the surface. Physically, the curvature parameter is inversely related to the radius of the cylinder, so increment in values of $\gamma$ reduces the radius of the cylinder, consequently the contact region between liquid and cylinder improves which causes less friction to the speed of fluid and hence the velocity profile increases. The variation of volume fraction of nanoparticles is given in Fig. 6. This figure depicts decline in velocity profile with the raise of nanoparticle volume fraction. The main reason of such decline is that as the values of nanoparticle volume fraction increases, the resistive force also increases which reduces the fluid’s flow speed due to which velocity decreases. Consequently, we can say that rise in values of nanoparticles volume fraction leads to drops the fluid velocity.

The consequences of varying quantities on the temperature profile have been presented in Figs. 7, 8, 9, 10, 11, 12, 13, 14. In Figs. 7 and 8, the influence of the radiation parameter *Rd* and volume fraction of nanoparticles ($\phi_{1} {,}\phi_{2}$) on the temperature profile are drawn up respectively. Temperature profile enhances with rising values of thermal radiations (*Rd*). Physically, greater values of *Rd* have dominant effects over conduction. Therefore, due to radiation good amount of heat released in the system which rises the temperature. Temperature profile shows increasing behavior with increment in $\phi_{1} ,\phi_{2}$. It has been analyzed that due to an increment in Fe_3_O_4_ nanoparticle volume proportion, the temperature of the fluid increases. The reason behind is the fact that the thermal characteristics of fluids are enhanced by adding and increasing the proportion of nanoparticles. Moreover, the addition of nanoparticles to the base fluid improves the capacity of the material for heat transfer which leads to increase the temperature profile.

Figure 9 drafts the consequences of curvature parameter $\gamma$ on temperature profile. Conduction is further dominated close to surface, so near the wall thickness of thermal boundary layer and temperature decreases and increases away from the cylinder surface. The reason for this behavior is that, increase in curvature enhances the rate of heat transfer from cylinder to the surface, thus temperature of the fluid drops near the surface and strengthen away from the surface. Figure 10 demonstrates the consequence of *Pr* on temperature field. It decays considering substantial values of *Pr*, because *Pr* is fraction of mass diffusivity to thermal diffusivity therefore, increase in *Pr* slows down the heat diffusion rate which causes both temperature field and boundary layer thickness decays.

Figures 11, 12, 13, 14 shows the outcomes of space dependent parameter ($X^{*}$) and time dependent parameter ($Y^{*}$) for internal heat generation (positive values) and internal heat absorption (negative values) on temperature profile. The existence of heat generation (space and time dependent heat greater than zero) raises the temperature of the fluid by adding more heat to the system and decreases the thickness of thermal boundary layer. Also, for heat absorption (space and time dependent heat less than zero) take in heat from thermal boundary layer leading to drop the temperature profile.

Tables 3 and 4 shows the numerical values of the skin friction coefficient and heat transfer rate for different values of the involved physical parameters It is noted that skin friction coefficient increases by increasing the Hartmann number, while slip parameter decreases the shear stress on the surface. Also, the skin friction coefficient is inversely related to curvature of the stretching cylinder. Radiation and Prandlt number increases the rate of mass transfer on the surface. In Table 5 nomenclature is given.

**Table 3:** Numerical values of skin friction coefficient $- \frac{1}{{A_{1} }}f^{{\prime \prime }} \left( 0 \right)$.

*M*	*β*	γ	*Rd*	Pr	*ϕ*_1_, *ϕ*_2_	$- \frac{1}{{A_{1} }}f^{{\prime \prime }} \left( 0 \right)$
0	0.4	0.2	0.8	6.2	0.2	1.18309
2						1.65539
4						1.81153
6						1.89943
2	0.0	0.2	0.8	6.2	0.2	5.40617
	0.2					2.5069
	0.4					1.65539
	0.6					1.23934
2	0.4	0	0.8	6.2	0.2	1.62225
		0.3				1.6705
		0.6				1.71125
		0.9				1.74659
4	0.4	0.2	0.6	6.2	0	1.99326
					0.05	1.99326
					0.1	1.76277
					0.15	1.50842

**Table 4:** Numerical values of Nusselt number $- A_{5} \theta^{{\prime }} \left( 0 \right)$**.**

*M*	*β*	γ	*Rd*	Pr	*ϕ*_1_, *ϕ*_2_	$- A_{5} \theta^{\prime}\left( 0 \right)$
4	0.4	0.2	0	6.2	0.2	2.00336
			0.3			2.83816
			0.6			4.24755
			0.9			7.5965
4	0.4	0.2	0.6	4	0.2	4.02301
				5		4.11232
				6		4.22268
				7		4.3574
4	0.4	0.2	0.6	6.2	0	-5.49465
					0.05	-5.49465
					0.1	2.3497
					0.15	1.75107

**Table 5:** Nomenclature.

Nomenclature			
*u*, *v*	*x* and *r* components of velocity (ms^−1^)	**Greek symbol**	
*T*	Fluid temperature (K)	*ϕ*_1_, *ϕ*_2_	Nanoparticles volume fraction
*T*_*w*_	Wall temperature (K)	ρ	Fluid density (kgm^−3^)
$T_{\infty }$	Ambient temperature (K)	Ψ	Stream function
B_0_	Strength of magnetic field	μ	Dynamic viscosity (kgm^−1^ s^−1^)
*B*	Radius of the cylinder (m)	υ	Kinematic viscosity (m^2^ s^−1^)
*U*_*w*_	Stretching velocity (ms^−1^)	σ	Electric conductivity (Ω^−1^ m^−1^)
$\sigma^{*}$	Stefan-Boltzmann constant (Wm^−2^ K^−4^)	*c*_*p*_	Specific heat (Jkg^−1^ K)
$k^{*}$	Mean absorption coefficient	κ	Thermal conductivity (Wm^−1^ K^−1^)
X*	Space dependent heat	**Subscript**	
Y*	Time dependent heat	*f*	Base fluid
*q*_r_	Radiative heat flux (Wm^−2^)	*nf*	Nanofluid
*q*ʺʹ	Non-uniform heat flux	*hnf*	Hybrid Nanofluid
*M*	Magnetic field parameter	*s*_1_	First solid nanoparticle
Pr	Prandtl number	*s*_2_	Second solid nanoparticle
*Rd*	Radiation parameter		
*β*	Slip parameter		
*γ*	Curvature parameter		

## Conclusion

In this paper, we have presented the investigations of MHD hybrid nanofluid over a stretching cylinder. We have incorporated the effects of thermal radiation and non-uniform heat flux with velocity slip condition. The mathematical modeling is carried out by using the continuity, momentum and energy equation. A set of suitable transformations and non-dimensional variables have been utilized to transform the governing partial differential equations into set of non-dimensional ordinary differential equations, which are then solved numerically. Plots of several physical quantities have been prepared to get the right insight of the considered problem. It is observed that, for realistic values of the controlled parameters, we observe that the velocity of the fluid decreases as the Hartmann number, the slip parameter, and nanoparticles volume fraction increases; it increases away from the surface with increasing the curvature parameter. The temperature of the fluid increases as radiation parameter, nanoparticles volume fraction, and non-uniform heat source/sink parameters increases. For increasing curvature parameter, it decreases near the surface and increases away from the surface. The Prandtl number also decreases the temperature of the fluid. We noted that hybrid nanomaterial work efficiently in processes involving high temperatures. These includes solar energy, refrigeration systems, air conditioning applications, heat exchanger, coolants in machining and auto motives, transformer cooling, nuclear system etc.
